# Enhanced nutrition improves growth and increases blood adiponectin concentrations in very low birth weight infants

**DOI:** 10.3402/fnr.v60.33171

**Published:** 2016-12-01

**Authors:** Elin W. Blakstad, Sissel J. Moltu, Britt Nakstad, Marit B. Veierød, Kenneth Strømmen, Pétur B. Júlíusson, Astrid N. Almaas, Arild E. Rønnestad, Kristin Brække, Christian A. Drevon, Per O. Iversen

**Affiliations:** 1 Department of Pediatric and Adolescent Medicine, Akershus University Hospital, Lørenskog, Norway; 2 Institute of Clinical Medicine, Campus Ahus, University of Oslo, Oslo, Norway; 3 Department of Nutrition, Institute of Basic Medical Sciences, Faculty of Medicine, University of Oslo, Oslo, Norway; 4 Department of Neonatal Intensive Care, Women and Children's Division, Oslo University Hospital, Ullevål, Oslo, Norway; 5 Oslo Centre of Biostatistics and Epidemiology, Department of Biostatistics, Institute of Basic Medical Sciences, Faculty of Medicine, University of Oslo, Oslo, Norway; 6 Department of Neonatal Intensive Care, Division of Paediatric and Adolescent Medicine, Oslo University Hospital, Rikshospitalet, Oslo, Norway; 7 Department of Clinical Science, University of Bergen, Bergen, Norway

**Keywords:** premature, metabolic syndrome, leptin, adiponectin, IGF-1

## Abstract

**Background:**

Adequate nutrient supply is essential for optimal postnatal growth in very low birth weight (VLBW, birth weight<1,500 g) infants. Early growth may influence the risk of metabolic syndrome later in life.

**Objective:**

To evaluate growth and blood metabolic markers (adiponectin, leptin, and insulin-like growth factor-1 (IGF-1)) in VLBW infants participating in a randomized nutritional intervention study.

**Design:**

Fifty VLBW infants were randomized to an enhanced nutrient supply or a standard nutrient supply. Thirty-seven infants were evaluated with growth measurements until 2 years corrected age (CA). Metabolic markers were measured at birth and 5 months CA.

**Results:**

Weight gain and head growth were different in the two groups from birth to 2 years CA (weight gain: *p*_interaction_=0.006; head growth: *p*_interaction_=0.002). The intervention group improved their growth *z*-scores after birth, whereas the control group had a pronounced decline, followed by an increase and caught up with the intervention group after discharge. At 5 months CA, adiponectin concentrations were higher in the intervention group and correlated with weight gain before term (*r*=0.35) and nutrient supply (0.35≤*r*≤0.45). Leptin concentrations correlated with weight gain after term and IGF-1 concentrations with length growth before and after term and head growth after term (0.36≤*r*≤0.53).

**Conclusion:**

Enhanced nutrient supply improved early postnatal growth and may have prevented rapid catch-up growth later in infancy. Adiponectin concentration at 5 months CA was higher in the intervention group and correlated positively with early weight gain and nutrient supply. Early nutrition and growth may affect metabolic markers in infancy.

Clinical Trial Registration (ClinicalTrials.gov) no.: NCT01103219

Early supply of adequate nutrient supply to premature infants is essential for optimal postnatal growth and development (1). Increased nutritional support reduces the incidence of growth failure (2–4) and improves neurodevelopmental outcomes (5, 6). Despite improvements in nutritional care, with evidence of increased growth velocity among very low birth weight (VLBW, birth weight (BW)<1,500 g) infants, a recent study showed that 50% of VLBW infants still exhibit postnatal growth failure (7). Moreover, it has been reported that premature infants have higher fat mass and increased risk of adverse cardiovascular and metabolic outcomes later in life as compared to full-term infants (8–10). How and when postnatal nutrition and growth may affect these outcomes are not fully understood (11). Few have studied the associations between growth and metabolic markers in VLBW infants fed according to recent nutritional recommendations.

Adiponectin and leptin, secreted by adipose tissues, are examples of relevant metabolic markers that can be measured in blood (12, 13). In adults, adiponectin concentrations are bell shaped: low with very small adipose depots, increasing with enhancing adipose tissues, and decreasing with obesity (14). Adiponectin levels in newborns are higher than in adults and correlate positively with BW (15, 16). During the first weeks of life, adiponectin levels increase before they decrease to lower values at 1 year of age (17). Premature infants have lower adiponectin levels as compared to term infants, but the levels increase markedly postnatally (18, 19). Reduced adiponectin levels are associated with increased risk of obesity and metabolic syndrome in children (20).

Leptin levels correlate positively with body fat mass, and leptin is important in the regulation of food intake and energy expenditure (12). In term infants, leptin levels correlate positively with birth weight, gain in fat mass, and body mass index (BMI) (17, 21). Leptin levels increase after birth until 6 months, but showed a trend to return to birth levels by 1 year of age (17).

Insulin-like growth factor-1 (IGF-1) promotes cell growth, inhibits apoptosis, and stimulates glucose uptake and protein synthesis (22). This polypeptide is related to nutritional status (22) and associated with postnatal growth in term and preterm infants (23–25).

In a randomized controlled trial (RCT), we investigated the effects of enhanced versus standard nutrient supply to VLBW infants (3). Enhanced nutrition resulted in improved growth during the first weeks of life (3). In this study, we evaluated growth during the first 2 years of life and blood concentrations of adiponectin, leptin, and IGF-1 at birth and at 5 months corrected age (CA). We hypothesized that enhanced nutrition has an effect on metabolic markers and promotes steady growth patterns.

## Methods

### The original RCT

**Fig. 1:**
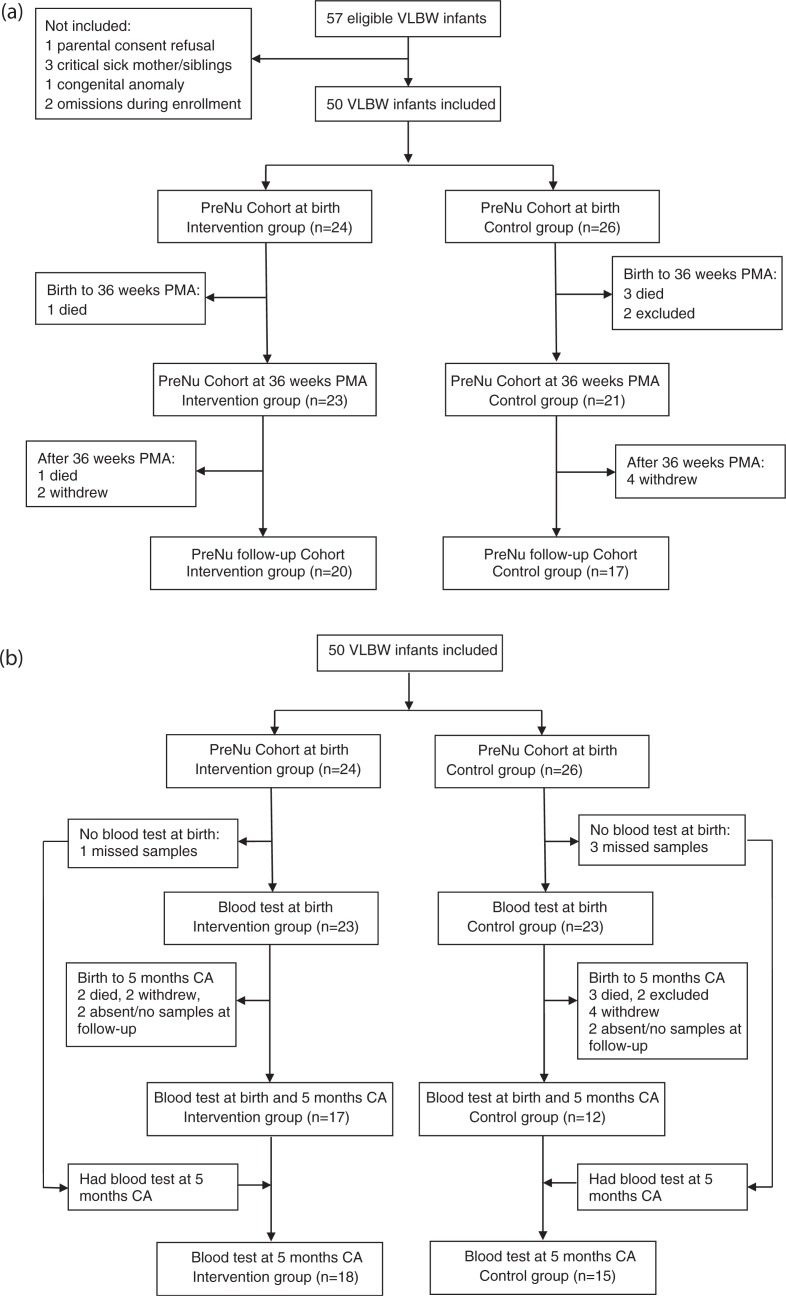
(a) Flowchart of study participants with anthropometric data. During the first weeks of life, four infants died and two infants were excluded due to critical illness and congenital heart disease. After discharge, one infant died and six withdrew from the study. (b) Flowchart of study participants with blood tests sampled. Four infants did not have blood samples at birth. During the first weeks of life, four infants died and two infants were excluded due to critical illness and congenital heart disease. After discharge, one infant died and six withdrew. Four infants did not attend/did not have samples collected at follow-up. The four infants without blood tests at birth had tests at 5 months CA. CA=corrected age, PMA=post menstrual age, VLBW=very low birth weight.

This is a follow-up of an open RCT conducted at Akershus and Oslo University Hospitals in 2010. The study was approved by the Regional Committee for Medical and Health Research Ethics (3). VLBW infants were included after informed parental consent was obtained. Exclusion criteria, estimation of sample size, and the randomization procedure have been described previously (3). The primary endpoint of the RCT was to reduce the proportion of infants discharged as growth restricted from 60 to 40%. After inclusion of 50 infants, a pre-planned safety analysis revealed increased occurrence of late-onset septicemia in the intervention group and further enrollment was stopped (26). Thus, the original study included 50 infants (hereby referred to as the PreNu cohort). Thirty-seven infants (hereby referred to as the PreNu follow-up cohort) were followed up with anthropometric measurements during the first 2 years of life (Fig. 1a).

### Nutrition intervention

The nutrition protocol has previously been described in detail (3, 26). Fifty infants were randomized to either an intervention group (enhanced nutrient supply) or a control group (standard nutrient supply). The intervention group started with 3.5 g/kg/d of amino acids and 2.0 g/kg/d of fish oil-containing lipid emulsion (SMOF; Fresenius Kabi, Norway), and the control group started with 2.0 g/kg/d of amino acids and 0.5 g/kg/d of lipid emulsion (Clinoleic; Baxter, Norway). Enteral feeding was given from the first day of life, mother's milk or banked human donor milk, increased equally with time and fortified with Nutriprem^®^ (Nutricia, Norway) in both groups. Additional supplements for the intervention group were amino acids (0.6 g Complete Amino Acid Mix^®^, Nutricia, Norway/100 mL human milk), long-chain polyunsaturated fatty acids (PUFA), 60 mg/kg/d each of docosahexaenoic acid (DHA) and arachidonic acid (AA) (Formulaic, Martek, USA), and 1,500 µg/kg/d of vitamin A (Aas Laboratory, Norway). The total amount of energy and protein on full enteral feeding was estimated to be 166 kcal/kg/d and 4.4 g/kg/d to the intervention group and 146 kcal/kg/d and 3.6 g/kg/d to the control group.

When breastfeeding, the intervention group infants received additional ‘protein-shots’ (1.6 g Complete Amino Acid Mix/20 mL human milk) per kg (maximum of 3/day). If they were not breastfed, the infants got a special preterm formula (Enfalac Premature, 81 kcal/100 mL, Mead Johnson, Norway). The intervention ended at 52 weeks postmenstrual age (PMA) and/or when the body weight reached 5.5 kg. Parenteral and enteral nutrient intakes were registered daily and calculated during the first 4 weeks of life.

### Assessments of growth

Small for gestational age (SGA) was defined as weight <10th percentile. Length, weight, and head circumference (HC) were monitored until 2 years CA. From birth to discharge, growth data were collected from the hospital growth charts. After discharge, the infants were followed at local health care centers where trained nurses monitored their growth. At 5 months CA, body weights were registered at a follow-up visit at the hospital. We used the weights collected at the 5 month follow-up. For the remaining growth data we used the measurements closest to selected time points during the first 2 years of life. Non-sex-specific *z*-scores for length and HC were calculated by Fenton growth charts until 36 weeks PMA (www.ucalgary.ca/fenton). Sex-specific weight *z*-scores were obtained by Skjærven's growth charts until 36 weeks (27). Norwegian growth data were used as a reference to calculate *z*-scores from term (minimum gestational age [GA] 38 weeks+6 days) until 2 years CA (28).

### Measurements of metabolic markers

The blood concentrations of adiponectin, leptin, and IGF-1 were assessed within the first 2 days of life (hereafter referred to as at birth) and at approximately 5 months CA using dried blood spots (DBS; Vitas Analytical Services, Oslo, Norway). At both sampling points, whole blood was collected from central/peripheral catheters or from a heel/finger prick, added to a special filter card, and stored at −80°C until analyses. Analyses of metabolic markers were performed with AM-296, AM-263, and AM-248 quantification for adiponectin, leptin, and IGF-1, respectively, in DBS using ELISA (www.vitas.no).

## Statistics

### Analyses of clinical characteristics and nutrient intake

To study group differences in clinical characteristics and nutritional intakes, we used a Student's *t*-test or a Mann–Whitney's U test for continuous variables and a chi-square or Fisher's exact test for categorical variables. Results are presented as means (ranges), percentages, or medians (interquartile ranges, IQRs).

### Analyses of growth

To assess growth and growth differences between the two groups from birth to 2 years of age, we used a linear mixed model procedure (covariance structured model for repeated observations with unstructured variance). The dependent variables were *z*-scores for weight, length, or HC, and fixed factors were group and time. We tested for interaction between group and time (i.e. whether the two groups changed over time in different ways).

### Analyses of metabolic markers

To study blood concentrations of adiponectin, leptin, and IGF-1, the results were log_e_ (ln) transformed to normalize data. Statistical analyses were performed on transformed data, and the back-transformed means (geometric means) with confidence intervals are presented. Group differences in metabolic markers at birth were evaluated with Student's *t*-test. Analysis of covariance (29) was used to study differences in change in metabolic markers from birth to 5 months CA. Infants without data of metabolic markers at birth were excluded from the analysis at 5 months CA. Due to a strong correlation between birth adiponectin concentrations and BW (i.e. the problem of collinearity), we did not adjust for BW when comparing adiponectin concentrations between the groups. For leptin and IGF-1, adjusting for BW did not change the conclusions of the analyses (data not shown).

Pearson's correlation coefficient (*r*) was calculated to study associations between metabolic markers and clinical characteristic at birth, between metabolic markers and parameters of growth, and between metabolic markers and specific nutrients. All test results at birth (*n*=46) and at 5 months CA (*n*=33) were used in the correlation analyses. Significance was assumed for *p*<0.05. Statistical analyses were performed by SPSS, version 22 (SPSS, Chicago, IL, USA).

## Results

### Study sample

Forty-four infants (PreNu cohort) had anthropometric data at 36 weeks GA, and 37 infants (the PreNu follow-up cohort) were followed with anthropometric measurements throughout the first 2 years of life (Fig. 1a). Forty-six infants (23 in each group) had blood samples collected at birth (Fig. 1b). Thirty-three infants had blood samples at 5 months CA, but 4 of these infants did not have blood samples at birth, leaving 29 infants with samples at both birth and 5 months CA (Fig. 1b). Clinical characteristics were similar between the two groups, except in the PreNu follow-up cohort where mean BW was significantly lower in the intervention group compared to the controls (Table 1).

**Table 1:** Clinical characteristics at birth and calculated daily nutrient intakes during the first 4 weeks of life

	PreNu cohort			PreNu follow-up cohort		
		
	Intervention (*n*=24)	Control (*n*=26)	*p*	Intervention (*n*=20)	Control (*n*=17)	*p*
GA at birth (weeks), mean (range)	28.1 (25.0–33.6)	28.1 (23.0–32.6)	0.94	28.0 (25.0–33.6)	28.6 (24.0–32.6)	0.54
Sex, boys, *n* (%)	15 (63%)	15 (58%)	0.73	12 (60%)	12 (71%)	0.51
SGA, *n* (%)	11 (46%)	6 (23%)	0.09	10 (50%)	3 (18%)	0.08
Birth weight (g), mean (range)	940 (460–1311)	1,035 (426–1414)	0.19	912 (460–1244)	1,122 (571–1,414)	<0.01
Birth weight *z*-score, mean (range)	−1.29 (−3.51–0.70)	−0.95 (−2.64–0.03)	0.22	−1.34 (−3.51–0.70)	−0.87 (−1.90– −0.15)	0.14
Energy, kcal/kg/d, median (IQR)	139 (128–145)^a^	126 (121–128)^b^	<0.001	139 (129–145)	126 (121–127)	<0.001
Protein, g/kg/d, median (IQR)	4.0 (3.9–4.2)^a^	3.2 (3.1–3.3)^b^	<0.001	4.1 (3.9–4.2)	3.2 (3.1–3.3)	<0.001
AA, mg/kg/d, median (IQR)	68 (57–73)^a^	24 (23–25)^b^	<0.001	68 (58–72)	24 (23–24)	<0.001
DHA, mg/kg/d, median (IQR)	87 (81–91)^a^	36 (34–38)^b^	<0.001	87 (81–91)	36 (34–37)	<0.001

Data are presented as mean (range), median (interquartile range, IQR), or frequency (%). AA=arachidonic acid, DHA=docosahexaenoic acid, GA=gestational age, SGA=small for gestational age.

a *n*=23

b *n*=21.

### Weight, length and HC z-scores

**Fig. 2:**
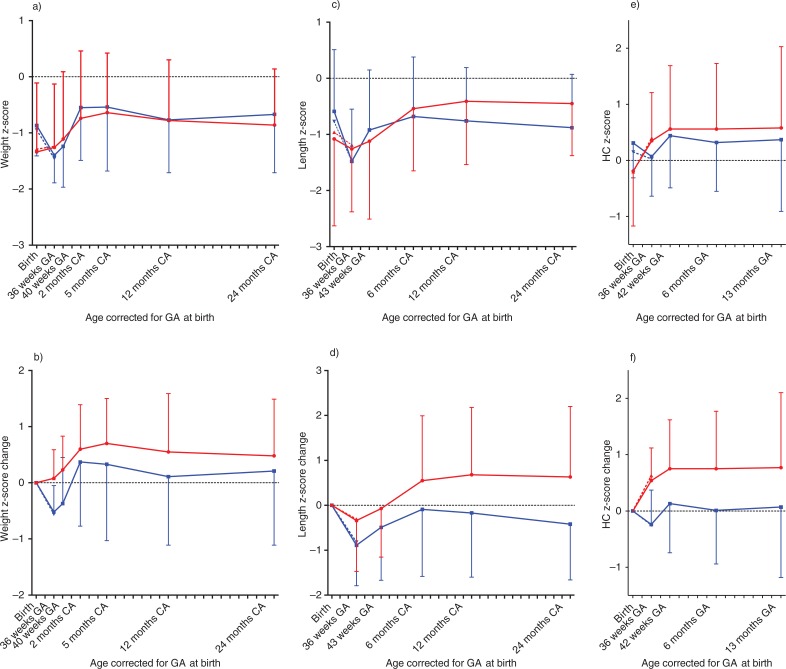
Anthropometric data from birth to 2 years corrected age. *z*-scores and *z*-score changes for weight, length and head circumference (HC). Panel a, c, e: mean (SD) *z*-scores for each group at selected time points; panel b, d, f: change in *z*-scores from birth, estimated from means from each group at specific time points. Error bars represents standard deviations. Whole lines represent the PreNu follow-up cohort (*n*=35–37). Red and blue dotted lines represent the PreNu cohort (*n*=49–50 at birth and *n*=40–44 at 36 weeks). GA=gestational age.

There was a significant difference between the groups in weight gain from birth to 24 months CA (*p*_interaction_=0.006) (Fig. 2a). However, the difference was most evident from birth to 40 weeks GA, when the control group had a pronounced decline in weight *z*-scores followed by an increase in growth and subsequent catch-up with the intervention group (Fig. 2a and b) after discharge. Mean weight *z*-scores remained negative for both groups, and 27% of the infants were growth restricted (weight <10th percentile [28]) at 2 years CA (intervention group 30%, control group 24%; *p*=0.73).

There was no significant difference between the groups in length growth over time (*p*_interaction_=0.35) (Fig. 2c), but length *z*-scores changed significantly in both groups over time (*p*<0.001), and the change was most evident between birth and 36 weeks CA (*p*=0.001) (Fig. 2c and d). Mean *z*-scores remained negative and 25% of the infants had length *z*-scores below the 10th percentile (28) at 2 years CA (intervention 25%, control group 25%; *p*=1.00).

We observed a significant difference between the groups in HC growth over time (*p*_interaction_=0.002) (Fig. 2e). The intervention group had a positive change in *z*-scores between birth and 36 weeks PMA, whereas the control group had a marked decline. From 36 weeks PMA, mean HC *z*-scores in the control group also increased (Fig. 2e and f). Mean *z*-scores remained positive for both groups. However, at 13 months CA, 22% of the infants had *z*-scores under the 10th percentile (28) (intervention group 25%, control group 18%; *p*=0.70).

**Fig. 3:**
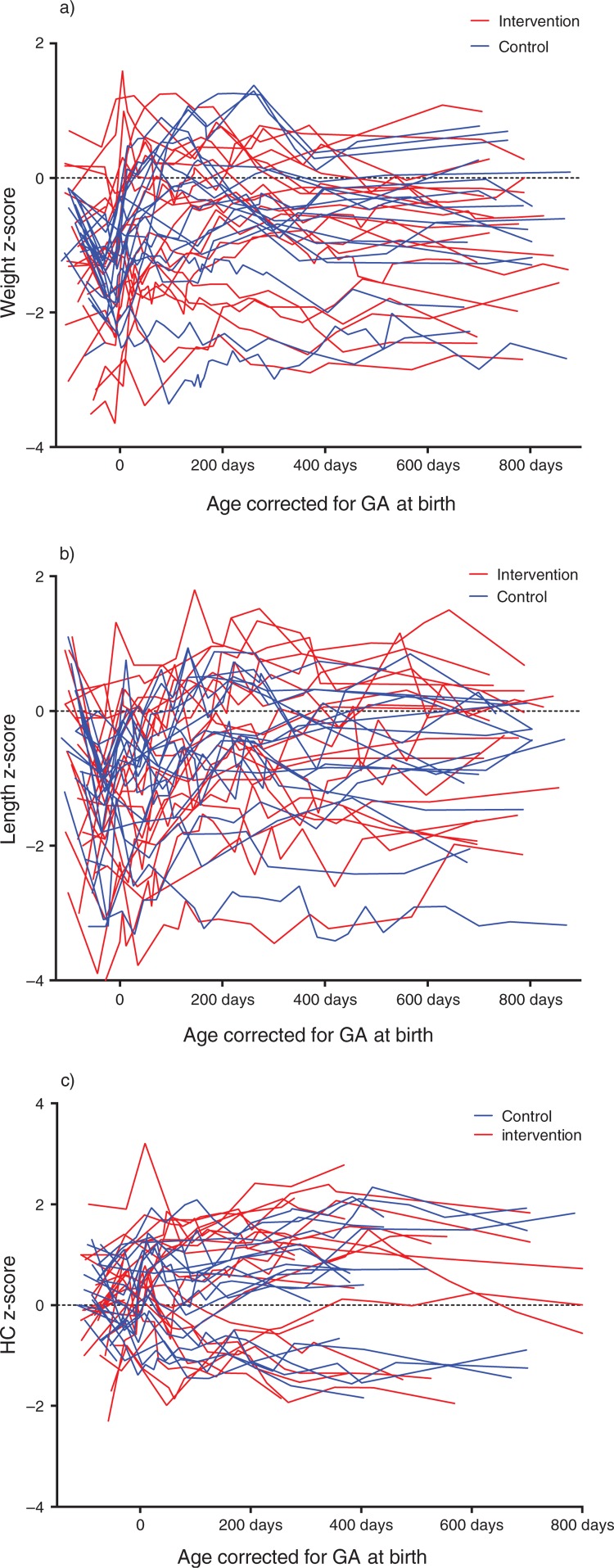
Individual growth profiles. Individual *z*-scores for weight (a), length (b), and head circumference (HC) (c). GA=gestational age.

Additional adjustment for SGA did not change the conclusions for weight, length, and HC growth (data not shown). One infant in the control group was diagnosed with a congenital heart defect causing heart failure and poor growth. Analysis without this infant did not change the conclusions for weight, length, or HC (data not shown). Individual growth profiles are presented in Fig. 3.

### Metabolic markers at birth and at 5 months CA

The two groups had similar mean (SD) age at blood sampling at birth (intervention group vs. control group: 0.2 [0.5] days vs. 0.4 [0.5] days chronological age [*p*=0.23]), and at 5 months CA (intervention group vs. control group: 155 (11) and 159 (13) days CA; *p*=0.25)).

There were no significant differences between the two groups in blood concentrations of adiponectin, leptin and IGF-1 at birth (Table 2). A few outliers were identified with higher blood concentrations of metabolic markers. Excluding these infants from the analysis did not change the results (data not shown).

**Table 2:** Concentrations of metabolic markers in the intervention and the control group at birth and at 5 months corrected age

	PreNu cohort at birth			PreNu follow-up cohort at birth			PreNu follow-up cohort at months CA		
			
	Intervention (*n*=23)	Control (*n*=23)	p*	Intervention (*n*=17)	Control (*n*=12)	p*	Intervention (*n*=17)	Control (*n*=12)	p**
Adiponectin (µg/mL)	3.9 (2.9–5.4)	4.7 (3.3–6.5)	0.44	3.8 (2.6–5.5)	5.4 (3.8–7.6)	0.17	16.5 (14.2–19.1)	13.7 (11.8–15.9)	0.03
Leptin (pg/mL)	2.0 (1.9–2.2)	2.1 (1.9–2.3)	0.64	2.1 (1.9–2.3)	2.0 (1.8–2.3)	0.79	2.3 (2.1–2.6)	2.8 (2.1–3.9)	0.19
IGF-1 (nmol/L)	27.1 (26.0–28.2)	27.7 (26.2–29.2)	0.54	27.0 (25.6–28.4)	28.8 (26.6–31.1)	0.13	29.8 (28.6–31.0)	28.7 (26.8–30.8)	0.12

Data are presented as geometric mean (95% CI).

* Student's t-test.

** Analysis of covariance. CA=corrected age, IGF-1=insulin-like growth factor 1.

At 5 months CA, the intervention group had significantly higher adiponectin concentrations compared to controls (*p*=0.03), while no significant differences were observed for leptin and IGF-1 (Table 2). One infant in the control group had a very high leptin value at 5 months CA. After excluding this outlier, there was still no significant difference between the groups (*p*=0.46).

There were no differences in any of the metabolic markers at birth or at 5 months CA when comparing infants born SGA to infants born appropriate for gestational age (AGA) (Table 3).

**Table 3:** Concentrations of metabolic markers in infants born appropriate for gestational age (AGA) and small for gestational age (SGA)

	PreNu cohort at birth			PreNu follow-up cohort at birth			PreNu follow-up cohort at months CA		
			
	AGA (*n*=31)	SGA (*n*=15)	p*	AGA (*n*=19)	SGA (*n*=10)	p*	AGA (*n*=19)	SGA (*n*=10)	p**
Adiponectin (µg/mL)	4.4 (3.5–5.5)	4.1 (2.3–7.2)	0.82	4.8 (3.8–6.1)	3.7 (2.0–7.1)	0.43	15.0 (13.1–17.2)	15.7 (12.8–19.3)	0.51
Leptin (pg/mL)	2.1 (1.9–2.3)	2.0 (1.8–2.3)	0.79	2.0 (1.9–2.2)	2.2 (1.8–2.5)	0.39	2.5 (2.1–3.1)	2.5 (2.1–3.1)	0.99
IGF-1 (nmol/L)	27.7 (26.5–28.9)	26.8 (25.2–28.4)	0.34	27.8 (26.3–29.4)	27.4 (25.3–29.8)	0.77	29.0 (27.7–30.5)	29.9 (28.4–31.5)	0.36

Data are presented as geometric mean (95% CI).

* Student's t-test.

** Analysis of covariance. CA=corrected age, IGF-1=insulin-like growth factor 1.

### Correlations between metabolic markers and birth characteristics, growth and nutrient supply

Strong positive correlations were observed between blood concentrations of adiponectin at birth and weight, length, HC, and GA at birth (0.63≤*r*≤0.76) (Table 4). No significant correlations were found between leptin and IGF-1 concentrations at birth and anthropometric measures or GA (0.04≤*r*≤0.20), or between any of the metabolic markers and birth *z*-scores (−0.01≤*r*≤0.18).

**Table 4:** Correlations between metabolic markers and anthropometric data and gestational age at birth

	Adiponectin (*n*=46)		Leptin (*n*=46)		IGF-1 (*n*=46)	
			
	*r*	*p*	*r*	*p*	*r*	*p*
BW	0.76	<0.001	0.05	0.77	0.14	0.36
BW *z*-scores	0.06	0.69	−0.01	0.91	0.04	0.80
Birth length^a^	0.64	<0.001	0.20	0.19	0.15	0.32
Birth length *z*-scores^a^	−0.01	0.93	0.14	0.37	0.05	0.73
Birth HC	0.74	<0.001	0.13	0.39	0.20	0.19
Birth HC *z*-scores	0.17	0.26	0.09	0.56	0.18	0.24
GA birth	0.63	<0.001	0.04	0.78	0.11	0.46

BW=birth weight, GA=gestational age, HC=head circumference, IGF-1=insulin-like growth factor 1, *r*=Pearson's correlation coefficient, Δ*z*-score=*z*-score change

a *n*=45.

Blood concentration of adiponectin at 5 months CA correlated positively with weight gain from birth to 40 weeks PMA (*r*=0.35) (Table 5). Leptin concentrations correlated positively with weight gain from 40 weeks PMA to 5 months CA (*r*=0.53). IGF-1 concentration correlated positively with length growth from birth to 43 weeks PMA (*r*=0.50) and from 43 weeks PMA to 6 months CA (*r*=0.36) and HC growth from 42 weeks PMA until 6 months CA (*r*=0.51). Adiponectin correlated positively with the first 4 weeks mean supply of protein, DHA, and AA and with first week mean supply of energy, protein, DHA, and AA (0.35≤*r*≤0.45) (Table 5).

**Table 5:** Correlations between metabolic markers at 5 months corrected age and growth and nutrient supply

	Adiponectin (*n*=33)		Leptin (*n*=33)		IGF-1 (*n*=33)	
			
	*r*	*p*	*r*	*p*	*r*	*p*
Δ*z*-score weight, birth to term (40 weeks PMA)	0.35	0.04	−0.11	0.55	0.19	0.29
Δ*z*-score weight, term (40 weeks PMA) to 5 months CA	−0.25	0.17	0.53	0.001	0.32	0.07
Δ*z*-score length, birth to term (43 weeks PMA)^a^	0.09	0.65	−0.10	0.61	0.50	0.004
Δ*z*-score length, term (43 weeks PMA) to 6 months CA^a^	−0.14	0.46	0.26	0.16	0.36	0.05
Δ*z*-score HC, birth to term (42 weeks PMA)	−0.01	0.95	−0.23	0.21	0.10	0.58
Δ*z*-score HC, term (42 weeks PMA) to 6 months CA	−0.02	0.92	0.15	0.40	0.51	0.002
First 4 weeks mean protein supply	0.35	0.05	−0.26	0.15	0.10	0.59
First 4 weeks mean energy supply	0.28	0.11	−0.28	0.12	0.28	0.12
First 4 weeks mean DHA supply	0.37	0.03	−0.28	0.11	0.16	0.37
First 4 weeks mean AA supply	0.35	0.05	−0.26	0.15	0.18	0.31
First week mean protein supply	0.41	0.02	−0.33	0.06	0.18	0.32
First week mean energy supply	0.45	0.008	−0.28	0.11	0.34	0.05
First week mean DHA supply	0.39	0.03	−0.28	0.12	0.19	0.29
First week mean AA supply	0.41	0.02	−0.20	0.27	0.27	0.13

AA=arachidonic acid, CA=corrected age, DHA=docosahexaenoic acid, HC=head circumference, IGF-1=insulin-like growth factor 1, PMA=post menstrual age, *r*=Pearson's correlation coefficient, Δ*z*-score=*z*-score change

a *n*=31.

## Discussion

Our data demonstrate that enhanced nutrient supply improves growth in VLBW infants. After the initial physiological weight loss, the intervention group infants showed early catch-up growth followed by a more steady growth pattern up to 2 years CA. The control group infants had a pronounced initial decline in *z*-scores followed by catch-up growth mainly after neonatal hospitalization. As far as we are aware, this is the first RCT to examine whether enhanced nutrient supply and improved early growth have an effect on metabolic markers in VLBW infants. Blood adiponectin concentrations correlated positively with anthropometric measures at birth, weight gain from birth to term, and supply of nutrients. At 5 months CA, adiponectin concentrations were higher in the intervention group compared to the controls.

Enhanced nutrient supply limited the prolonged and severe decline in postnatal growth, which was observed in the control group. This is in line with Senterre and Rigo's (4) study, which also demonstrated that postnatal weight loss can be limited by reducing postnatal nutrient deficiencies. For premature infants, early growth failure appears to be especially harmful in certain periods of organ development. The brain is extremely vulnerable to deprivation of specific nutrients between 24 and 42 weeks PMA (30). Impaired postnatal growth has negative effects on neurodevelopment in premature infants (31), and increased early protein and energy supply has been associated with higher cognitive scores (5). Belfort et al. (6) reported a positive association between increased BMI and weight gain before term and improved neurodevelopment in preterm infants. After term weight gain, but not weight gain out of proportion to length, was beneficial for neurodevelopment (6).

In our study, growth restriction (<10th percentile) was still prevalent through the first years of life in almost a quarter of the infants. Mean *z*-scores for weight and length remained negative throughout the first 2 years, whereas mean HC *z*-scores were positive for both groups at 13 months CA, suggesting that head growth was prioritized before weight and length growth. Others also report that head growth recovers before weight and length growth. Westerberg et al. (32) identified that VLBW children were smaller and lighter than their peers, but that head growth normalized after 2 months. Ramel et al. (33) demonstrated that head growth recovered by 4 months CA, whereas linear growth continued to be suppressed at 24 months.

We observed that blood adiponectin concentrations increased from birth to 5 months CA in both groups, supporting what has previously been reported by others (19, 34, 35). Moreover, we found that infants on enhanced nutrient supply had a greater increase in adiponectin concentrations as compared to the infants on standard nutrient supply. This is the first study to report a difference in adiponectin concentrations between two groups of premature infants with different nutrient supplies. Other studies of premature infants have described positive associations between adiponectin and postnatal growth. Hansen-Pupp et al. (19) demonstrated a positive correlation between adiponectin concentrations during the first 3 weeks of life and growth variables at 35 weeks GA. Saito et al. (35) observed a positive correlation between weight gain and change in adiponectin concentrations. Siahanidou et al. (36) reported that adiponectin concentration at discharge correlated positively with the last week weight gain.

We found that the first week supply of protein, energy, and essential fatty acids correlated positively with adiponectin concentrations at 5 months CA. The correlations remained positive for the first 4 weeks’ mean values of protein and PUFAs, but not for energy. Hansen-Pupp et al. (19) reported that adiponectin concentrations correlated positively with energy intake, but the correlation disappeared after adjusting for GA at birth. Yoshida et al. (34) reported that adiponectin concentration at term correlated inversely with age to regain birth weight, but not with energy supply. Notably, the nutrient supply in these two studies was much lower than in our intervention. Siahanidou et al. (36) found higher levels of adiponectin concentrations in infants fed formula containing long-chain PUFAs, but did not observe an effect of last week energy supply.

In line with other studies (19, 35, 37, 38), we found that adiponectin concentrations at birth had a strong positive correlation with anthropometric measures and GA at birth. We did not observe differences in adiponectin concentrations at birth or at 5 months CA between infants born SGA or AGA. In contrast to this, one study of term and preterm infants reported lower adiponectin concentrations 3–13 days postnatally in infants born SGA compared with AGA (38). Saito et al. (35) found no effect of SGA status on adiponectin concentrations at birth, but demonstrated lower adiponectin concentrations at term-equivalent age in SGA born infants. Other studies also report that preterm SGA born infants have consistently lower adiponectin concentrations postnatally until term (19) and at discharge (36). Even though it was not significant, the majority of the SGA born infants in our study was randomized to the intervention group. It is possible that enhanced nutrient supply and subsequent improved early growth had a positive impact on adiponectin concentrations in the SGA infants, promoting similar concentrations as the AGA infants.

We demonstrated a positive correlation between blood leptin concentrations at 5 months and weight gain from term to 5 months CA. This is supported by van Poelje et al. (21) who identified that weight *z*-scores gain from term to 6 months CA resulted in higher fat mass and higher leptin concentrations at 6 months CA, indicating that leptin is a marker of body fat mass during the first 6 months after term. Bozzola et al. (17) identified higher leptin concentrations in AGA versus SGA term born infants at birth and comparable levels at 1st, 6th and 12th month of age. The authors suggested the increased leptin levels were due to increased adipose tissue.

In this study, we observed positive correlations between IGF-1 concentrations and length growth (birth to 6 months) and head growth (birth to 43 weeks). A recent review pointed out that IGF-1 concentrations correlate differently with growth before and after term-equivalent age in preterm infants (22). Hansen-Pupp et al. (24) reported that IGF-1 concentrations correlated positively with weight gain at an early postnatal age in preterm infants. Brain volumes in preterm infants at term age were positively associated with postnatal IGF-1 concentrations (39). Van de Lagemaat et al. (25) reported that both preceding length and weight growth were positively associated with IGF-1 at term, 3 and 6 months CA. After term age, studies show contradictive results in relation to IGF-1 concentrations and correlations with subsequent growth (22).

There have been concerns that enhanced nutrient supply and excessive postnatal growth increase the risk of developing adiposity and metabolic syndrome later in life in preterm infants (8–10). Metabolic markers measured in children have been associated with adiposity and adverse metabolic outcomes (20, 40). The association between metabolic markers in infancy and later risk of obesity and metabolic syndrome is, however, not clear. Increase in leptin concentrations between term and birth may mirror an increase in fat deposition. The increase in fat depositions could be a result of rapid catch-up growth after the period of growth retardation similar to what we observed in our control group. Furthermore, excessive fat deposition is a possible risk factor for metabolic syndrome. The infants in the intervention group did not experience the prominent growth retardation and avoided the subsequent period of rapid catch-up growth. We speculate that the higher adiponectin concentration in the intervention group could be an indication of a healthier growth pattern. However, this needs to be investigated further.

Our study has limitations. The sample size is small (3) and differences may not have been detected due to low statistical power. Despite randomization, the intervention group had a significantly lower BW than the control group (3). BW *z*-scores were non-significantly lower in the intervention group. *Z*-scores were used when comparing growth over time. The lower BW in the intervention group may have had an undetected impact on the potential of growth and changes in metabolic markers. Also, we did not fully adjust for confounding factors because of low power. However, the sample size is similar to other studies examining metabolic markers in premature infants (17, 19, 24, 34, 35). Another limitation was the variety in number of anthropometric measurers for each participant. This was due to differences in lengths of hospital stay and number of visits to the local health care institutions. The anthropometric measurement closest to each selected time points was used to calculate mean values. Also, we would have preferred to use one growth chart, but there are no common Norwegian growth charts for weight, length, and HC for premature infants from birth through early childhood. We justify the use of different growth charts, because we compared differences between two groups and not to the general population. In this study, we did not include information about maternal nutrition, BMI, smoking habits, and morbidities. These factors might influence nutrition and growth. We used DBS in our analysis to reduce blood volumes and still keep the sensitivity of the assays (38, 41). Most other studies have used plasma or serum. This makes it difficult to directly compare our values of the metabolic markers with levels reported by others.

## Conclusions

Enhanced nutrient supply improved postnatal growth with a marked reduction in infants subjected to postnatal growth restriction. The correction of early growth might have prevented the need for catch-up growth close to term age, where excessive growth often is dominated by increase in adipose tissue and a subsequent increase in the risk of metabolic syndrome. We observed a higher adiponectin concentration in the intervention group at 5 months CA and a positive correlation between adiponectin concentrations and growth before term. Early nutrition and growth may affect metabolic markers in infancy. We did not demonstrate any changes in the metabolic markers suggesting elevated risk of metabolic syndrome among the infants receiving enhanced nutrient supply.
