# Corrigendum

**DOI:** 10.29219/fnr.v62.3346

**Published:** 2018-12-27

Blakstad EW, Moltu SJ, Nakstad B, Veierød MB, Strømmen K, Julıusson PB, et al. Enhanced nutrition improves growth and increases blood adiponectin concentrations in very low birth weight infants. Food Nutr Res 2016; 60: 33171. https://doi.org/10.3402/fnr.v60.33171

In the version of this article published online, the leptin levels in Tables 2 and 3 were reported in the wrong units as pg/mL (picogram/mL). The correct unit should be ng/mL (nanogram/mL). This has now been corrected and hereby updated as follows: ‘In Tables 2 and 3, the concentrations of leptin are reported as ng/mL (nanogram/mL).’

This correction does not alter the study’s findings of significance or overall interpretation of the study results. The authors regret for any inconvenience caused.

